# Impact and cost-effectiveness of rotavirus vaccination in Bangladesh

**DOI:** 10.1016/j.vaccine.2017.05.087

**Published:** 2017-07-13

**Authors:** Clint Pecenka, Umesh Parashar, Jacqueline E. Tate, Jahangir A.M. Khan, Devin Groman, Stephen Chacko, Md Shamsuzzaman, Andrew Clark, Deborah Atherly

**Affiliations:** a PATH, 2201 Westlake Ave, Suite 200, Seattle, WA 98121, USA; b Centers for Disease Control and Prevention, 1600 Clifton Rd, Atlanta, GA 30329, USA; c Liverpool School of Tropical Medicine, Pembroke Place, Liverpool L3 5QA, United Kingdom; d WHO Country Office, 10 Gulshan Avenue, Gulshan-1, Dhaka 1212, Bangladesh; e National Immunization Program of Bangladesh, Directorate General of Health Services (DGHS), EPI Bhaban, Mohakhali, Dhaka 1212, Bangladesh; f London School of Hygiene & Tropical Medicine, Keppel Street, London WC1E 7HT, United Kingdom

**Keywords:** Bangladesh, Cost-effectiveness, Rotavirus, Vaccination, DALY

## Abstract

**Introduction:**

Diarrheal disease is a leading cause of child mortality globally, and rotavirus is responsible for more than a third of those deaths. Despite substantial decreases, the number of rotavirus deaths in children under five was 215,000 per year in 2013. Of these deaths, approximately 41% occurred in Asia and 3% of those in Bangladesh. While Bangladesh has yet to introduce rotavirus vaccination, the country applied for Gavi support and plans to introduce it in 2018. This analysis evaluates the impact and cost-effectiveness of rotavirus vaccination in Bangladesh and provides estimates of the costs of the vaccination program to help inform decision-makers and international partners.

**Methods:**

This analysis used Pan American Health Organization’s TRIVAC model (version 2.0) to examine nationwide introduction of two-dose rotavirus vaccination in 2017, compared to no vaccination. Three mortality scenarios (low, high, and midpoint) were assessed. Benefits and costs were examined from the societal perspective over ten successive birth cohorts with a 3% discount rate. Model inputs were locally acquired and complemented by internationally validated estimates.

**Results:**

Over ten years, rotavirus vaccination would prevent 4000 deaths, nearly 500,000 hospitalizations and 3 million outpatient visits in the base scenario. With a Gavi subsidy, cost/disability adjusted life year (DALY) ratios ranged from $58/DALY to $142/DALY averted. Without a Gavi subsidy and a vaccine price of $2.19 per dose, cost/DALY ratios ranged from $615/DALY to $1514/DALY averted.

**Conclusion:**

The discounted cost per DALY averted was less than the GDP per capita for nearly all scenarios considered, indicating that a routine rotavirus vaccination program is highly likely to be cost-effective. Even in a low mortality setting with no Gavi subsidy, rotavirus vaccination would be cost-effective. These estimates exclude the herd immunity benefits of vaccination, so represent a conservative estimate of the cost-effectiveness of rotavirus vaccination in Bangladesh.

## 1 Introduction

Diarrheal disease is one of the leading causes of child mortality globally, and rotavirus is responsible for more than a third of those deaths [1], [2], [3]. While there is some variation in global mortality estimates by source, rotavirus mortality has fallen dramatically over the past two decades. In 2000, the number of deaths due to rotavirus disease was estimated to be 518,000 per year in children under five years of age. In 2013, this number decreased to 215,000 rotavirus deaths per year in children under five years of age. Of these deaths, 121,000 occurred in Sub-Saharan Africa and 89,000 occurred in Asia. As many as 2700 of these deaths are estimated to occur in Bangladesh [2], [4]. While deaths are an important component of rotavirus burden, there are additional health and economic consequences due to rotavirus disease.

Currently, there are two World Health Organization (WHO) pre-qualified rotavirus vaccines available globally to help reduce the burden of rotavirus disease. These two vaccines are Rotarix® (manufactured by GlaxoSmithKline), administered as a two-dose schedule, and RotaTeq® (manufactured by Merck & Co., Inc.), administered as a three-dose schedule. According to WHO recommendations, rotavirus vaccines should be introduced into every country’s national immunization program, particularly those where diarrhea is a leading cause of child death. Consistent with this recommendation, more than 80 countries have introduced rotavirus vaccination [5]. Gavi, the Vaccine Alliance also supports rotavirus vaccination by subsidizing the cost of vaccination in eligible countries [6]. Despite the WHO recommendation, Gavi support and large declines in global mortality, substantial mortality, morbidity, and economic burden due to rotavirus disease remain [7].

Bangladesh has played a leading role in building the evidence base for rotavirus vaccination, as well as other interventions to combat diarrheal disease (i.e., oral rehydration salts) [8], [9]. In 2016, Bangladesh applied for Gavi support for rotavirus vaccination and plans to introduce vaccination in 2018. This analysis evaluates the impact and cost-effectiveness of rotavirus vaccination in Bangladesh and provides estimates of the costs of the vaccination program to help inform decision-makers in Bangladesh and international partners.

## 2 Materials and methods

This analysis examines the cost-effectiveness of a routine infant rotavirus vaccination program in Bangladesh compared to no vaccination. We examine nationwide introduction of a two-dose rotavirus vaccine beginning in 2017. Benefits and costs are examined from the societal perspective over ten successive birth cohorts. Costs and benefits are discounted at 3% per annum. All monetary units are adjusted to 2016 USD. Key outputs of the analysis include: deaths averted; disability adjusted life years (DALYs) averted; cases averted; inpatient visits averted; outpatient visits averted; informal “visits” averted; and health costs averted as a result of rotavirus vaccination. Additional outputs include total cost of vaccination program and cost/DALY averted.

This analysis tracks disease events and costs in ten vaccinated cohorts over the first five years of their life. During this time, they may or may not get rotavirus disease. If they acquire rotavirus, it can be either non-severe or severe. In either case, treatment for rotavirus disease can be sought as an inpatient (facility-based setting) or as an outpatient (facility-based or informal setting). A facility-based setting could be a hospital or clinic and an informal setting could include a faith-based healer or the acquisition of oral rehydration salts. Non-severe disease results in recovery with or without informal or outpatient care. Severe disease results in recovery or death with or without informal or inpatient care.

### 2.1 Model

This analysis uses Version 2.0 of the TRIVAC model. This Excel-based model was developed by researchers from the London School of Hygiene and Tropical Medicine (LSHTM) with support from Pan American Health Organization’s (PAHO’s) ProVac Initiative. The model is designed to be used at the country-level to conduct cost-effectiveness analyses for three vaccines: Rotavirus vaccine, Pneumococcal conjugate vaccine, and *Haemophilus influenza* type b and provides a consistent and transparent framework for comparing the impact and cost-effectiveness of these vaccines [10]. The model has been widely used across the world to evaluate the cost-effectiveness of these vaccines [11], [12], [13], [14], [15], [16]. The model input parameters include: demographics, burden of disease, vaccine schedule, vaccine efficacy, vaccine coverage, vaccine costs, health service utilization, and health service costs. More detail on input parameters and values is included below.

### 2.2 Demographic data

Data on the number of live births and life expectancy at birth were gathered from the United Nations Populations Division [17]. Infant and child mortality data in Bangladesh were taken from the United Nations Inter-agency Group for Child Mortality Estimation [18]. The annual rate of reduction in infant and child mortality over the 2005–2015 period was used to project infant and child mortality rates through 2026.

### 2.3 Disease burden

#### 2.3.1 Incidence and severity

The incidence of rotavirus in the under-five population is estimated to be 10,000 cases per 100,000 children. This value is based on a systematic review and meta-analysis by Bilcke et al. [19]. It is also corroborated by Zaman et al. which found an incidence of rotavirus gastroenteritis of 9,500/100,000 person years in the control arm of a RotaTeq trial in Bangladesh and in Vietnam [8]. Platts-Mills et al. note that 13% of all cause *diarrhea* episodes were severe, and subsequent unpublished analysis indicates that 27% of rotavirus episodes were severe in the same study [20], [21]. This is a conservative estimate of rotavirus severity relative to other studies in the region [8]. Severity is defined by the duration and number of loose stools; and duration of vomiting, dehydration, and fever.

#### 2.3.2 Mortality

There is substantial divergence in rotavirus mortality estimates in Bangladesh. Some estimate fewer than 1000 deaths per year [3] while others estimate over 2700 deaths per year [4]. To account for this divergence, we examine a midpoint mortality scenario with 1850 deaths from rotavirus in children under five per annum prior to vaccination. We also include scenarios with 1000 and 2700 rotavirus deaths in children under five prior to vaccination. A more extensive set of disease burden parameters is included in Table 1.

**Table 1:** Input parameters for estimating disease burden.

Parameter	Estimate	Source/s
**Annual incidence per 100,000 aged 1**–**59** m**onths:**		
Rotavirus (non-severe) cases	7300	Assumption, based on [19], [20], [21]
Rotavirus (severe) cases	2700	Assumption, based on [19], [20], [21]
Rotavirus deaths (low, mid, high estimates)^a^	1000,1850, 2700	[2], [3]

**Disability weight for DALY calculations**		
Rotavirus (non-severe) cases	0.188	Data from supplementary tables in [22]
Rotavirus (severe) cases	0.247	Data from supplementary tables in [22]

**Mean duration of illness (in days)**		
Rotavirus (non-severe) cases	6	Assumption
Rotavirus (severe) cases	6	Assumption

**Age distribution of disease cases and deaths**		
<3 months:	0.5%	[23]
3–5 months:	6.1%	[23]
6–8 months:	21.8%	[23]
9–11 months:	22.7%	[23]
12–23 months:	46.0%	[23]
24–35 months:	2.7%	[23]
36–47 months:	0.1%	[23]
48–59 months:	0.0%	[23]

a These estimates were calculated using the case fatality ratio. These calculations are aligned with estimates from the IHME, CDC, and WHO as referenced in text.

### 2.4 Vaccine coverage and efficacy

Vaccine coverage is high in Bangladesh. DTP1 is 97% and the second dose is interpolated as 95.5% based on DTP3 coverage of 94% [24]. Vaccine efficacy for severe and non-severe disease in the first year following vaccination is 48% and 45.2%, respectively. Vaccine efficacy decreases by 36% per year.^1^ These values are based on an unpublished analysis of a Rotarix® trial in Bangladesh [25]. We assume single dose efficacy is half that of the two dose course. We exclude any indirect benefit of vaccination, i.e. herd effects.

### 2.5 Vaccine price and delivery cost

We model the vaccine price using Gavi’s projection of Bangladesh’s co-financing shares as they increase over time. Using a Gavi price of $2.19 per dose results in a vaccine price for Bangladesh of $0.16 in 2017 and this increases by 15% a year to $0.55 by 2026 [26], [27]. We compare this to a vaccine price of $2.19 per dose, without a Gavi subsidy. We assume an additional 3% and 2% of the vaccine cost for handling and delivery, respectively. We also assume 5% vaccine wastage.

We initially, and conservatively, estimated the delivery cost as $0.80 per dose. This estimate is an average cost per dose delivered across the relevant Expanded Program on Immunization (EPI) cost categories for 2017 in the 2014–2018 cMYP [28]. Rotavirus relevant expenditure categories include personnel; maintenance and overhead; short term training; information, education, communication (IEC) and social mobilization; disease surveillance; program management; other routine recurrent costs; vehicles; and other capital equipment. These costs were allocated to rotavirus vaccines and other vaccines on a per dose basis. New cold chain investments were allocated to rotavirus vaccines and other new vaccines by volume, and the cost per dose of these capital investments was evenly distributed over ten years. Commodities, transport, campaign, and shared health systems costs were excluded. Upon consultation with local experts, we revised our initial estimate to include only half of personnel costs. The rationale is that the incremental labor costs associated with an additional vaccine are expected to be much lower than the average labor cost per dose. Using a lower, but still conservative, labor estimate yields a per dose cost of delivery of $0.54. We explore this variable in the sensitivity analysis.

### 2.6 Health service utilization and costs

Per Das et al., 88% of children under five receive care outside the home for diarrhea [29]. However, the 2014 Demographic Health Survey reports that approximately 36% of children under five receive treatment from a facility or formal provider [30]. To account for the population that seeks care informally and to capture these informal costs, we differentiate cases into those that seek formal or informal care. If 88% of children with diarrhea receive formal or informal care and 36% of those seeking treatment receive formal care, this implies 41% (i.e. 0.36/0.88) of those seeking care will receive care in a facility. The remaining treatment seekers will seek care in an informal setting. This health service utilization data is combined with incidence data to determine the population that seeks care for rotavirus disease.

Rotavirus cases generate costs borne by households and providers, these costs are detailed in Table 2. Households will incur costs in the case of informal, formal outpatient, or formal inpatient care. Providers (e.g. the government) will incur costs for formal outpatient or formal inpatient care. We first discuss informal and formal outpatient costs for households and providers and then proceed to discuss inpatient costs for households and providers.

**Table 2:** Input parameters for estimating health service costs (all costs are presented in 2016 USD).

Parameter	Estimate	Source/s
**Government cost per visit**		
*Non-severe rotavirus cases*		
Facility (outpatient)	$1.88	[31]

*Severe rotavirus cases*		
Facility (inpatient)	$11.41	[33]

**Household cost per visit**		
*Non-severe rotavirus cases*		
Informal	$1.17	[31]
Facility (outpatient)	$1.39	[31]

*Severe rotavirus cases*		
Informal	$1.17	[31]
Facility (inpatient)	$51.21	[33]

Household costs associated with all informal treatment are estimated from Das et al. and sum to $1.17 [31].^2^ Household costs associated with formal outpatient care are estimated from the same source and total $1.39. These informal and outpatient cost estimates exclude lost income. Provider costs are zero in the case of informal care and total $1.88 for formal outpatient care based on estimates from Das et al. While the TRIVAC model differentiates between household and provider costs, Das et al. do not make this distinction. To avoid double counting, we allocate costs by category and allocate them to the household or provider in an attempt to adequately represent the costs borne by each group. Estimates of provider costs may be conservative as they are gathered from a study of household medical expenditure. Any provider costs that are not passed on to patients will be underestimated but would have a small impact on results. While imperfect, our estimates are in the range of those reported in Rheingans et al. increasing our confidence in our estimates [32].

Inpatient rotavirus costs were estimated from Ahmed et al. and total $51.21 for households and $11.41 for providers [33]. Inpatient household costs include both direct medical costs as well as indirect costs such as lost wages. All cost inputs were adjusted to 2016 USD using the period average official exchange rate and the consumer price index [34], [35].

## 3 Results

We present three scenarios that correspond to three distinct mortality estimates including a low estimate, high estimate, and a midpoint estimate [2], [3], [4]. The scenarios are detailed in Table 3 and illustrate the impact of rotavirus vaccination *over ten years* beginning in 2017. All model inputs are consistent across the scenarios with the exception of the case fatality rate for severe disease. Variation in the case fatality rate is used to influence pre-vaccination rotavirus mortality in the model. In each scenario we use a vaccine price per dose that accounts for a Gavi subsidy and increases over time from $0.16 in 2017 to $0.55 by 2026. We also examine the full cost of the vaccine at $2.19 per dose.

**Table 3:** Key model outputs by scenario.

	Midpoint mortality scenario	Low mortality scenario (IHME)	High mortality scenario (WHO/CDC)
Baseline rotavirus deaths (2017)	1850	980	2700
Baseline rotavirus admissions (2017)	160,000	160,000	160,000
Baseline rotavirus cases (2017)	1.5 million	1.5 million	1.5 million

**Model output with vaccination over 10** y**ears, benefits and costs discounted**			
Deaths averted	3900	2100	5800
DALYs averted	130,000	74,000	183,000
Cases averted	3.9 million	3.9 million	3.9 million
Inpatient visits averted	450,000	450,000	450,000
Outpatient visits averted	1.2 million	1.2 million	1.2 million
Informal “visits” averted	1.7 million	1.7 million	1.7 million
Health costs averted (government/societal)	$7.0/$33.7 million	$7.0/$33.7 million	$7.0/$33.7 million
Cost of vaccination program with and w/o Gavi subsidy (excludes health savings)	$44.3/$146.3 million	$44.3/$146.3 million	$44.3/$146.3 million
Cost/DALY averted with and w/o Gavi subsidy	$82/$871	$142/$1514	$58/$615
Cost/death averted with and w/o Gavi subsidy	$2689/$28,593	$5098/$54,207	$1840/$19,563
Cost/case averted with and w/o Gavi subsidy	$3/$29	$3/$29	$3/$29

With a Gavi subsidy, cost/DALY ratio’s ranged from $58/DALY averted in the high mortality scenario to $142/DALY averted in the low mortality scenario. Without a Gavi subsidy and a vaccine price of $2.19 per dose, the cost/DALY ratio is substantially higher, ranging from $615/DALY averted in the high mortality scenario to $1514/DALY averted in the low mortality scenario. Cost per death and cases averted are also contained in the table. In each scenario, rotavirus vaccination will avert thousands of deaths, DALYs, millions of cases and visits, as well as millions in health costs while also incurring millions of vaccination program costs. To put government costs in context, the net cost of the vaccine program in 2017 (with annualized introduction costs) is approximately one and a half percent of EPI program expenditure [28].

**Fig. 1:**
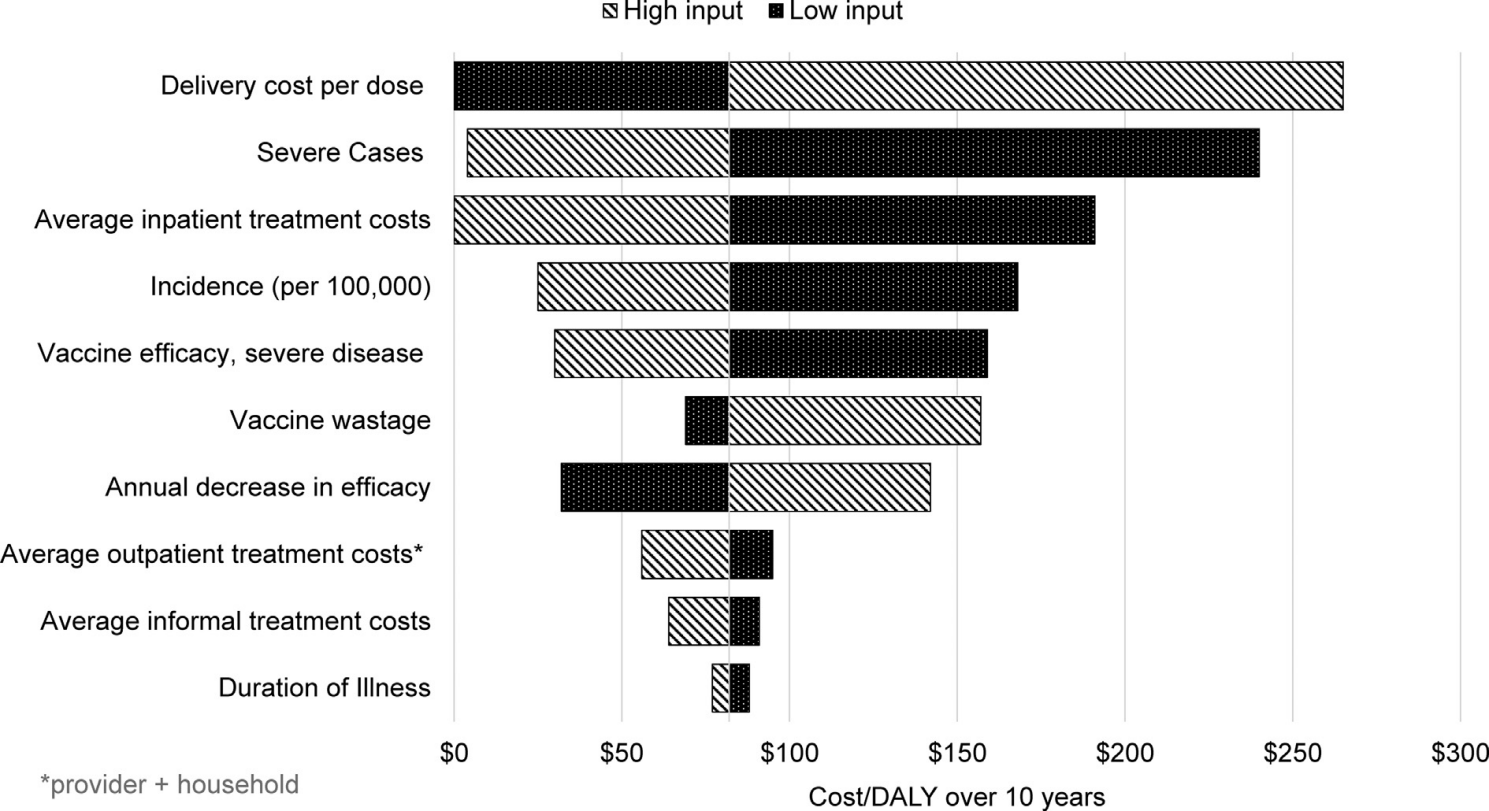
One-way sensitivity analysis of cost per DALY over 10 Years.

In addition to the three mortality scenarios presented above, we conducted a one way sensitivity analysis of the midpoint mortality scenario (incorporating the Gavi vaccine subsidy) to understand how variation in key input variables would impact results. Table 4 contains the base, low, and high data inputs. The impact these varying inputs have on the results is then represented in the tornado diagram (Fig. 1) that follows.

**Table 4:** Sensitivity analysis of midpoint mortality scenario.^a^

Input variable	Base input value	Low input value	High input value
Incidence (per 100,000)	10,000	8000	12,000
Severe cases	27%	17%	37%
Vaccine effectiveness, severe disease	48%	38%	58%
Vaccine wastage	5%	0%	20%
Annual decrease in effectiveness	36%	16%	56%
Delivery cost per dose	$0.54	$0.25	$1.00
Average inpatient treatment costs (provider + household)	$62.62	$31.31	$125.24
Average outpatient treatment costs (provider + household)	$3.27	$1.64	$6.54
Average informal treatment costs	$1.17	$0.59	$2.34
Duration of illness (non-severe and severe)	6 days	2 days	10 days

a Note that the variation in input values relative to the base input is not uniform. Also note that an increase in an input value may cause the cost-effectiveness ratio to decrease while an increase in another input value may cause an increase in the cost-effectiveness ratio.

The delivery cost per dose, the share of severe cases, and inpatient treatment costs have the largest impact on the results, given the range of inputs varied here (Fig. 1). At the low end of the delivery cost range and the high end of the severe cases and inpatient treatment cost range, rotavirus vaccination is cost saving. In no case does the cost/DALY exceed $275.

## 4 Discussion

Rotavirus vaccination would substantially reduce mortality, illness, and costs (including out of pocket costs) associated with rotavirus disease, and this is accounting only for the direct effects so these estimates are conservative. By almost any measure, rotavirus vaccination is highly cost-effective with a Gavi subsidy. Without a Gavi subsidy, the cost-effectiveness ratio is of the same magnitude as Bangladesh’s per capita income. Per capita income thresholds have often been used as a measure of cost-effectiveness, but there are limitations to that approach [36]. Ideally, cost-effectiveness should be included as an input into a transparent decision-making process alongside other considerations like budget impact, sustainability, feasibility, and equity [37]. Some countries have developed their own thresholds over time, and the authors of this work see value in a country specific approach [37]. However, no such threshold exists in Bangladesh. If per capita income thresholds were applied using Bangladesh’s 2015 per capita GNI of $1190, rotavirus vaccination would be highly cost-effective in five of six scenarios, and cost-effective in the “low mortality, no Gavi subsidy” scenario [38]. This result is consistent with results from other low resource countries with a high rotavirus burden [11], [12], [13], [39]. Importantly, this analysis also demonstrates that rotavirus vaccination can be cost-effective outside of high mortality contexts and absent Gavi support. This supports the argument that other countries in Asia may wish to consider rotavirus vaccination as a cost-effective intervention even in the absence of a high mortality burden or Gavi support. These results suggest that factors other than mortality (e.g., the cost of care that can be averted) will be an increasingly important rationale for introducing rotavirus vaccination for the countries in the region that have yet to introduce vaccination. In Bangladesh, the sensitivity analysis shows that averted inpatient expenditures are important. This includes out of pocket expenditures which are estimated to be over 60% of Bangladesh’s total health spending [40].

This study is a collaboration between international researchers, local policy makers, and vaccine program staff. Many of the data inputs were locally acquired and complemented by internationally validated estimates. As such, this study benefited from international rotavirus expertise as well as the strong evidence base developed in Bangladesh. One limitation of this analysis is the lack of detailed data on the marginal cost of vaccine delivery. However, engagement with local partners allowed us to coalesce around delivery cost estimates that made use of available data and also corresponded to local perspectives. While local data and expertise strengthened this analysis, it is important to note that this is a prospective analysis meaning that these are estimates of future benefits and costs rather than measurable retrospective outcomes. The TRIVAC model is a decision support tool that was critical in completing this analysis and provided a framework for country-level engagement regarding both data inputs and results. TRIVAC also provides an avenue to build local health economic capacity, not just for those engaged in data collection and modelling, but also decision-making.

Bangladesh recently made the decision to introduce rotavirus vaccination and has applied for Gavi support. This analysis played a role in informing international partners and local decision makers of the benefits and costs of rotavirus vaccination in Bangladesh. Cost-effectiveness analyses, alongside budget impact analyses, are playing an increasingly important role in guiding local and international decision-making. Given the strong results presented here, it is likely that other countries in the region may also find rotavirus vaccination to be both cost-effective and affordable.

## Author contributions

CP helped conceptualize the study, led data collection, analysis, drafting, and revision. AC, UP, JT, JK contributed data, helped conceptualize the study, interpreted results, and contributed to article drafting.

DG undertook components of the analysis and contributed to the drafting of the manuscript.

SC and MS helped conceptualize the study, contributed data and provided an essential link between the study and policy questions.

DA helped conceptualize the study and provided senior scientific support and oversight of the project.

All authors revised and approved of the final version of this article.

## Source of funding

This work was supported by the Bill & Melinda Gates Foundation, Seattle, WA [Grant No. OPP1053539 and OPP1147721].

## Conflict of interest statement

The authors have no conflicts to declare.

## Disclaimer

The findings and conclusions in this report are those of the authors and do not necessarily represent the official position of the US Centers for Disease Control and Prevention**.**

## Footnotes

1 This is a relative decrease, not an absolute decrease.

2 Median costs before attending a facility are a proxy for the cost of informal care. Also note, Das et al. do not distinguish costs by disease severity. We utilize their median cost estimates so costs do not reflect inpatient care.
